# Frequency of SARS-COV-2 infection and COVID-19 vaccine uptake and protection among Syrian refugees: COVID-19 Vaccine among Syrian Refugees

**DOI:** 10.1186/s12879-024-09460-4

**Published:** 2024-06-08

**Authors:** Zeynep Meva Altaş, Mehmet Akif Sezerol

**Affiliations:** 1 Department of Public Health, Ümraniye District Health Directorate, Istanbul, 34764 Türkiye; 2 Epidemiology Program, Institute of Health Sciences, Istanbul Medipol University, Istanbul, 34815 Türkiye; 3 Department of Public Health, School of Medicine, Istanbul Medipol University, Istanbul, 34815 Türkiye; 4 Department of Public Health, Sultanbeyli District Health Directorate, Istanbul, 34935 Türkiye

**Keywords:** Refugee, Syrian, COVID-19 infection, Vaccination, Vaccine effectiveness

## Abstract

It is aimed to examine the frequency of COVID-19 disease, the rates of COVID-19 vaccination and the vaccine effectiveness (VE) among Syrian refugees. It is a retrospective cohort study. Syrian refugees aged 18 years and above registered to a family health center in Sultanbeyli district in Istanbul were included. Vaccine effectiveness were calculated for both Pfizer BioN-Tech and CoronaVac (Sinovac) vaccines. The data of 2586 Syrian people was evaluated in the study. The median age of the participants was 34.0 years (min:18.0; max: 90.0). Of the participants 58.4% (*n* = 1510) were female, 41.6% (*n* = 1076) were male. In our study of the refugees 15.7% had history of COVID-19 infection. Refugees having full vaccination with Biontech and Sinovac have a significantly lower COVID-19 infection rate than those without vaccination (HR = 8.687; *p* < 0.001). Adjusted VE for Biontech, Sinovac, and both were 89.2% (95.0% CI:83.3–93.1), 81.2% (95.0% CI:48.72–93.1) and 88.5% (95.0% CI:82.7–92.3), respectively. The results of the study highlight the importance of vaccinations against COVID-19 pandemic, since both vaccines were highly protective in refugees.

**Supplementary Information:**

The online version contains supplementary material available at 10.1186/s12879-024-09460-4.

## Introduction

The COVID-19 disease, which started in China at the end of 2019, became a pandemic that has affected the entire world since 2020 [1]. Disadvantaged groups such as the elderly, patients with chronic diseases, and refugees are at higher risk for COVID-19 infection. Consequently, COVID-19 can result in serious infections among refugees [2, 3]. According to a systematic review, the incidence of COVID-19 infection has been shown to be higher among migrants, refugees, and asylum seekers [4]. It was reported that refugees in Norway have higher COVID-19 infection and hospitalization rates than Norwegian-born people [5]. Compared to the general population, the risk of COVID-19 infection among refugees and asylum seekers in reception facilities is 2.5 to 3 times greater [6].

Vaccination is a simple, safe and effective way for protection against contagious diseases [7]. With the emergence of the COVID-19 pandemic, researches on vaccine development have accelerated. Vaccination is extremely important in disease control both for the health of individuals and for herd immunity [8]. Four authorized COVID-19 vaccines are available worldwide. These vaccines are: BNT162 (Pfizer BioN-Tech, New York, NY, USA), ChAdOx1 (AstraZeneca, Oxford, UK), mRNA1273 (Moderna, Cambridge, MA, USA), and Ad26.COV2-S (Johnson & Johnson, New Brunswick, NJ, USA). Additionally; other vaccines, such as BBIBP-CorV (Sinopharm, Beijing, China), CoronaVac (Sinovac, Beijing, China), Sputnik V (Gamaleya, Moscow, Russian), and CO-VAXIN (Bharat Biotech, Hydrabad, India), are also authorized vaccines in several countries [9]. According to the clinical researches; in the vaccinated groups, the frequency of COVID-19 infection decreases and clinical findings are presented milder in case of a COVID-19 infection [10]. Besides, vaccination provides protection against the development of severe forms of COVID-19 [11]. However, in a community based research in our country, about the half of the participants were hesitant about the COVID-19 vaccines [12]. In another study, nearly 30.0% of the participants had no intention for COVID-19 vaccination [13]. In a study from the literature, less than half of the participants believed that COVID-19 vaccination programs would be successful in the fight against COVID-19 [14].

Vaccination services against COVID-19 have started gradually in Türkiye as in other countries. At first; healthcare workers, elders and people with chronic diseases were vaccinated. Subsequently, vaccination activities were applied in all age groups. Since the end of June 2021, adults of all age groups have been granted free vaccination by the Ministry of Health. CoronaVac (Sinovac), Pfizer-BioNTech and Turkovac vaccines are currently in use in Türkiye and these vaccines can be administered free of charge to everyone, including refugees. Vaccines are available at family health centers, public hospitals, private hospitals, and refugee health centers. Access to the vaccine is free for refugees, as it is for the entire population. Moreover, people can be tested even at home through the filiation process.

Variable rates of vaccination among refugees depending on the country, and timing of the study since the beginning of the pandemic have been reported in researches [15–17]. It is thought that the vaccination levels of refugees may be lower than the society due to adaptation to social life and access to vaccines. In a study it was reported that new immigrants have lower influenza vaccination rates [18]. On the other hand, due to the fact that refugees live in more crowded environments and have low socio-economic levels, the risk of COVID-19 infection may be higher than the community [5].

There is a high number of Syrian people in Türkiye. According to the latest data of the Directorate of Migration Management dated 19.04.2023; 3.411.029 registered Syrian individuals are living in Türkiye [19]. To enhance the accessibility of preventive and basic healthcare services for Syrian refugees in our country, Migrant Health Centers (MHC) have been established in areas with significant refugees, affiliated with primary healthcare institutions. One noteworthy aspect of these centers is the employment of Syrian healthcare professionals to bridge the language gap for immigrant individuals. Syrian refugees residing in Türkiye receive cost-free healthcare services, including vaccinations, through the MHC’s, family health centers, or hospitals.

Since refugees are more vulnerable to COVID-19 infection, the evaluation of COVID-19 infection rates and vaccine effectiveness in refugees is needed. There are several studies on vaccine effectiveness, but none, to our knowledge, specifically focus on refugees. In this context, this study aims to evaluate the frequency of COVID-19 disease, the COVID-19 vaccination rates of Syrian refugees living in the Sultanbeyli district of Istanbul, and the effectiveness of Sinovac and Biontech vaccines in this population.

## Methods

### Research type, population, sample

The research is a retrospective cohort study. There were 4590 Syrian refugees registered with a family physician working at a family health center in the Sultanbeyli district. Of these Syrian refugees, 2004 were children. Thus, the study population consists of 2586 Syrian refugees aged 18 years and above registered with a family physician working at a family health center in the Sultanbeyli district. All Syrian refugees registered with a family physician in Sultanbeyli were assessed for COVID-19 tests they had undergone in public and private primary, secondary, and tertiary healthcare institutions. Self-tested patients were not included. All test results performed at primary, secondary, and tertiary healthcare institutions are entered into the same system. The District Health Directorate can see all test results. While conducting the study, no contact was made with the refugees; only the data from the family health centers’ system was analyzed.

There are 24 family health centers in the Sultanbeyli district [20]. All Syrian refugees registered with a family health center in Sultanbeyli were included in the study without calculating a sample size. Sultanbeyli, with 358,201 residents, is a district in Istanbul, Türkiye, and has the lowest socio-economic development index compared to other districts of Istanbul [21]. Additionally, a significant population of Syrian refugees resides in Sultanbeyli [22].

### Measures

**Fig. 1:**
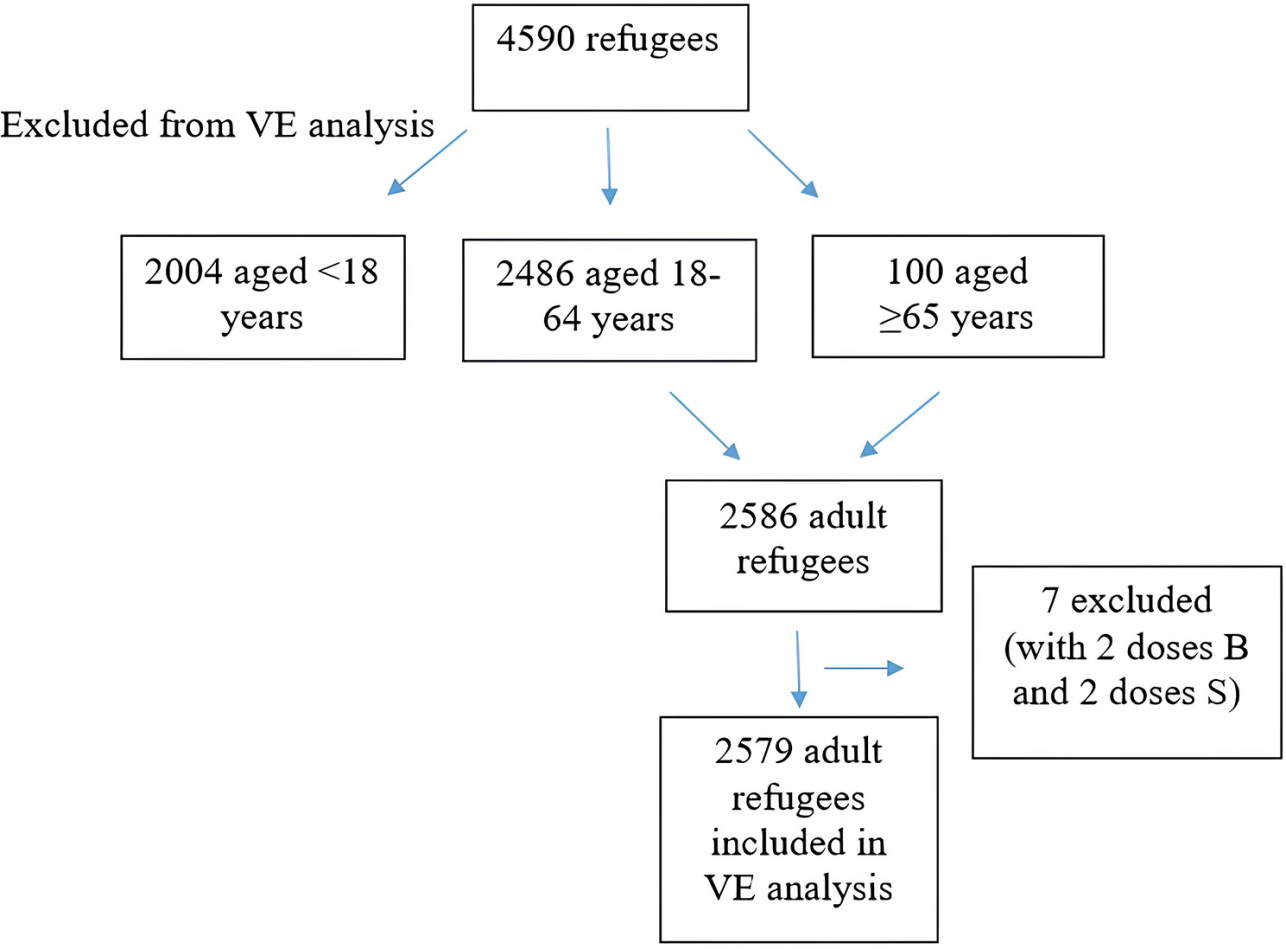
Participants included in vaccine effectiveness analysis. VE: Vaccine effectiveness, B:Biontech, S:Sinovac

The database includes information such as demographic information, COVID-19 vaccination dates and types, their COVID-19 infection status, and hospitalizations. Our study covers the data between dates March 2020 and August 2022. Individuals who had received at least two doses of the vaccine and had passed two weeks since their last dose were considered to be fully vaccinated [23]. Those who did not meet this criterion were in the unvaccinated group. COVID-19 infection rates of both groups were compared. Getting infected with COVID-19 two weeks after receiving two doses of the vaccine was considered as the case for analysis. Seven subjects with two doses of Biontech and two doses of Sinovac (a total of 4 doses) were not included in the vaccine effectiveness analysis. These seven individuals should be evaluated as a separate group since they are fully vaccinated with both vaccines. However, since 7 people are statistically very few in terms of forming a separate group, they were excluded from the vaccine effectiveness analysis (Fig. 1).

Those whose third dose was different from the other two doses were included in the effectiveness analysis for the two-dose vaccine. For example, a person vaccinated with 2 doses of Biontech and one dose of Sinovac was included in the Biontech vaccine effectiveness group. Vaccine effectiveness (VE) was calculated based on the incidences of COVID-19 infections within both groups to assess the relationship between vaccination status and COVID-19 infections. VE was calculated for both Biontech and Sinovac vaccines. The following formula was used for the calculation:

$$VE\, = \,1 - Relative\,Risk$$

VE = 1- (COVID-19 infection incidence in vaccinated arm): (COVID-19 infection incidence in unvaccinated arm)

### Statistical analysis

Statistical analysis of research data was made with SPSS 24.0 package program. In the study, median, minimum and maximum values, numbers (n) and percentages (%) were used for descriptive data. Conformity of continuous variables to normal distribution was examined by histograms, probability graphs and analytical methods. Fisher Exact test and Pearson chi-square tests were used to compare the categorical data. For the calculation of adjusted vaccine effectiveness (aVE), Cox regression analysis was used as multivariate analysis. In the Cox regression analysis, three models were computed. Age and gender were controlled for in all three models. In addition to age and gender, the first model adjusted for full vaccination status with the Biontech vaccine, the second model adjusted for full vaccination status with the Sinovac vaccine, and the third model adjusted for full vaccination status with either the Biontech or Sinovac vaccine. Hazard ratios were obtained from cox regression models. Adjusted VE was calculated as the formula written: aVE= [1-(1/HR (Hazard ratio) of full vaccination)]. The proportional hazards assumption and model fit was assessed by means of residual (Schoenfeld and Martingale) analysis. Statistical significance level was determined as *p* < 0.05.

### Ethics

The research was carried out in accordance with the Declaration of Helsinki Principles, no personal information that would reveal the private lives and/or identities of the participants was included, and the security of the data was ensured. The study was approved by Istanbul Medipol University Non-Interventional Clinical Research Ethics Committee with the decision number 824 on 27/09/2022. Since the study was conducted retrospectively, written informed consent was not obtained from the participants.

## Results

The data of 2586 Syrian people was evaluated in the study. The median age of the participants was 34.0 years (min:18.0; max: 90.0). The number of people between the age of 18–64 years and aged 65 years and above were 2486 (96.1%) and 100 (3.9%), respectively. Of the participants 58.4% (*n* = 1510) were female, 41.6% (*n* = 1076) were male. Ten (0.4%) participants were healthcare workers (Table 1).

**Table 1:** Sociodemographical features of the participants

Features	
Age (years), median (min-max)	34.0 (18.0–90.0)
Age groups, n (%)	
18–64 years	2486 (96.1)
65 years and above	100 (3.9)
Gender, n (%)	
Female	1510 (58.4)
Male	1076 (41.6)
Healthcare workers, n (%)	10 (0.4)

COVID-19 vaccination and infection status of the refugees were evaluated. Of the participants 36.4% (*n* = 941) had no vaccination. The percentages of refugees with single, two, three and four doses vaccines were 11.3% (*n* = 292), 47.1% (*n* = 1219), 4.9% (*n* = 126) and 0.3% (*n* = 8), respectively. The data about vaccination doses for the age groups are presented in Table 2.

**Table 2:** COVID-19 vaccination and infection status of the participants

	All ages	18–64 years	65 years ≥
COVID-19 vaccination, n (%)			
Single dose	292 (11.3)	285 (11.5)	7 (7.0)
Two doses	1219 (47.1)	1164 (46.8)	55 (55.0)
Three doses	126 (4.9)	110 (4.4)	16 (16.0)
Four doses	8 (0.3)	8 (0.3)	0 (0)
No vaccination	941 (36.4)	919 (37.0)	22 (22.0)
	All ages	18–64 years	65 years ≥
Full vaccination*, n (%)			
Biontech	1231 (47.6)	1185 (47.7)	46 (46.0)
Sinovac	113 (4.4)	89 (3.6)	24 (24.0)
	All ages	18–64 years	65 years ≥
COVID-19 infection, n (%)	405 (15.7)	387 (15.6)	18 (18.0)
COVID-19 reinfection, n (%)	16 (0.6)	15 (0.6)	1 (1.0)

* Full vaccination; defined as having at least two doses of the same type of vaccine. Those who received two doses of Sinovac and one dose of Biontech were considered to be fully vaccinated for the Sinovac vaccine

For the Biontech and Sinovac vaccines, the percentages of fully vaccinated (at least 2 doses of vaccine) refugees were 47.6% (*n* = 1231) and 4.4% (*n* = 113), respectively. Fully vaccination percentages according to the age groups are also shown in Table 2. Of the refugees 15.7% (*n* = 405) had history of COVID-19 infection. There were 14 participants hospitalized due to COVID-19 infection. One of the hospitalized patients were above 65 years). Intubation or mortality were not observed in hospitalized patients. All hospitalized patients were unvaccinated against COVID-19.

There were no participants received more than four doses of COVID-19 vaccine. Brand names of the COVID-19 vaccines for each doses were shown in Supplementary Fig. 2a and 2b.

Refugees vaccinated with Biontech and Sinovac have a significantly lower COVID-19 infection rate than those without vaccination. COVID-19 infection rates were found to be 1.9% in those who were fully vaccinated with Biontech, 3.5% in those who were fully vaccinated with Sinovac, and 16.4% in the unvaccinated (*p* < 0.001) (Table 3).

**Table 3:** The percentages of COVID-19 infection in vaccinated and unvaccinated refugees

	COVID-19 infection n (%)		*P* value*
	No	Yes	
Full vaccinated with Biontech	1208 (98,1)	23 (1.9)	<0.001
Full vaccinated with Sinovac	109 (96.5)	4 (3.5)	
Unvaccinated	1032 (83.6)	203 (16.4)	
All refugees (*n* = 2586)	2356 (91.1)	230 (8.9)	

*Significance is between unvaccinated and vaccinated with Biontech, and between unvaccinated and vaccinated with Sinovac

For Biontech vaccine and Sinovac vaccine; vaccine effectiveness were found as 88.6% and 78.5%, respectively. The calculations are presented below:

For Biontech vaccine:

$$VE\, = \,1 - \,\left[ {\left( {23/1231} \right)\,:\,\left( {203/1235} \right)} \right]\, = \,88.6\%$$

For Sinovac vaccine:

$$VE\, = \,1 - \,\left[ {\left( {4/113} \right)\,:\,\left( {203/1235} \right)} \right]\, = \,78.5\%$$

For the calculation of adjusted vaccine effectiveness, Cox regression analysis was used as multivariate analysis. COVID-19 infection was the dependet variable of the regression model. Getting infected with COVID-19 two weeks after two doses of vaccine was considered as the case for analysis. Age and gender were included in the Cox regression model in order to control their possible counfounding effects on COVID-19 infection. Three different models were created; each for the Biontech, Sinovac and both, respectively. In the first model for the Biontech vaccine, those who were fully vaccinated (at least two doses of vaccines) with Biontech and those who were not fully vaccinated were included in the model as an independent variable in addition to age and gender and compared in terms of COVID-19 infection. In other words, those fully vaccinated with Sinovac were not included in the first model. Similarly, Biontech fully vaccinated participants were not included in the second model for Sinovac. The follow-up period for the participants was considered to be the number of days (874 days) between the date the pandemic was declared and the date of our study ended. Adjusted VE for Biontech, Sinovac, and both vaccines were calculated as 89.2% (95.0% CI: 83.3–93.1), 81.2% (95.0% CI: 48.72–93.1) and 88.5% (95.0% CI: 82.7–92.3), respectively (Table 4).

**Table 4:** Multivariate analysis of the full vaccination

1. Model				
	P value	HR	95.0% CI for HR	
			Lower	Upper
Age	0.145	1.007	0.997	1.017
Gender*	0.352	0.882	0.676	1.149
Full vaccination-Biontech*	< 0.001	9.295	6.009	14.379
Adjusted VE (95.0% CI)	89.2% (83.3–93.1)			
2. Model				
	P value	HR	95.0% CI for HR	
			Lower	Upper
Age	0.064	1.010	0.999	1.020
Gender*	0.696	0.946	0.715	1.251
Full vaccination-Sinovac*	0.001	5.304	1.950	14.429
Adjusted VE (95.0% CI)	81.2% (48.72–93.1)			
3. Model				
Age	0.130	1.008	0.998	1.017
Gender*	0.435	0.901	0.692	1.172
Full vaccination-Biontech-Sinovac*	< 0.001	8.687	5.778	13.062
Adjusted VE (95.0% CI)	88.5% (82.7–92.3)			

VE: Vaccine effectiveness, HR: Hazard ratio. CI: Confidence interval

* For gender variable, male patients were the reference; for full vaccination, full vaccination was the reference value

* Adjusted VE was calculated as [1-(1/HR of full vaccination)]

## Discussion

In our study, of the participants 36.4% had no vaccination. Nearly half of the refugees had at least two doses of the COVID-19 vaccine. According to the data from the Ministry of Health of Türkiye, as of May 7, 2023, 93.4% of the population aged 18 years and above have received a single dose of the COVID-19 vaccine. The percentage of those vaccinated with two doses is 85.7% [24]. The COVID-19 vaccination rate among Syrian refugees is very low compared to the general population in Türkiye. The percentage of two-dose vaccination, which is considered full vaccination, is approximately 1.8 times higher in the general population than among refugees. Qualitative studies should be conducted to investigate the knowledge, thoughts, and attitudes about vaccines. Qualitative studies conducted with refugees from different educational levels, age groups, and economic statuses may provide a broader perspective. This may help in better understanding why the vaccination rates of Syrian refugees are low. In a study the willingness of refugees to receive COVID-19 vaccines is influenced by factors such as acquiring information from reliable sources and community engagement [25]. Considering the barriers against the vaccination, there is a need to organize vaccination campaigns for refugees, and to increase the encouragement and access to vaccines. Similar to our study, according to a study conducted in Norway, the percentage of individuals vaccinated with at least one dose of COVID-19 was 94% in Norwegian-born individuals, while this rate was reported as 73% in refugees. In a study conducted in the Canadian province of Alberta, COVID-19 vaccine coverage in refugees and the general population was reported as 78.2% and 76.0%, respectively [26]. The differences in the results of the studies may be due to the variations in the healthcare policies regarding refugees in the respective countries. The sociodemographic characteristics of refugees in different countries, their health literacy, the differences in health services, as well as other socioeconomic conditions in resident countries are among the reasons that can explain the differences between the studies.

Refugees have a higher risk of contracting COVID-19, experiencing the disease more severely, and having higher mortality rates compared to native-born individuals. Poorer housing conditions with higher overcrowding, higher use of public transport, living in areas with higher population density, language problems and other barriers to accessing health services are some factors causing higher infection and mortality rates among refugees [27]. In a cohort study conducted in Spain, involving cumulative incidence rate of COVID-19 among refugees was reported to be higher than among the host population [28]. However, a study conducted in Italy reports a similar prevalence of COVID-19 in refugees and native borns [29]. In our study of the refugees 15.7% had history of COVID-19 infection. In an epidemiological study conducted in our country, 15.7% of adults aged between 18-64 years and 26.5% of people aged 65 years and over stated that they had COVID-19 infection [30]. These studies conducted in our country are studies with small sample sizes and different methodologies. Another study conducted with Syrian the pregnant women in Türkiye, 4.2% of them had a COVID-19 infection during their pregnancy [31]. According to the March 2023 data of the Turkish Ministry of Health, there are a total of 17,232,066 COVID-19 cases [24]. According to the December 2022 data of the Türkiye Statistical Institute, the population of Türkiye has been reported as 85,279,553 [32]. As we know, there is no study comparing the cases of COVID-19 infection of refugees and local population in our country. Prospective studies with similar methodology in both populations are needed to evaluate the rates of COVID-19 infections in refugees and local populations.

In our study, there were 14 participants hospitalized due to COVID-19 infection. Only one of them was above 65 years of age. Since the percentage of unvaccinated refugees aged 65 years and over in our study was lower than 18–64 years old age group, the number of hospitalized patients in this age group may have been lower. Since all of the hospitalized people were unvaccinated; it can be said that the vaccine prevents severe infection as well as preventing infection at the age of 65 years and over. Another possibility is; it can be interpreted that the population aged 65 years and over, who is at risk of having a severe infection, was infected and became immune or died in the early stages of the pandemic, until the date of the study.

In our study, refugees vaccinated with Biontech and Sinovac had a significantly lower COVID-19 infection rate than those without vaccination. Similarly, according to Canadian data, among COVID-19 cases, 40.8% were unvaccinated, whereas 32.3% had completed their primary vaccine series. Among those hospitalized, the percentage of unvaccinated individuals is more than twice as high as those who have completed their primary vaccine series. According to the same data, the rate of mortality among the unvaccinated is about three times that of the vaccinated [33].

In our study, vaccine effectivenesses were found to be 88.6% and 78.5%, for Biontech and Sinovac vaccines respectively. Adjusted VE for Biontech, Sinovac, and both were 89.2%, 81.2% and 88.5%, respectively. In a study, after two months from Biontech vaccine, adjusted vaccine effectiveness was reported to be 81.3% in adults [34]. Another study reported the VE for Biontech as 86.9%. This effectiveness decreased to 43.3% after 6 months [35]. Moreover, the Biontech vaccine effectiveness has been reported to be 93.0% in people aged 16 years and older in Israel [36]. According to the population-based study conducted in adults aged 20–59 years in Hong Kong, the efficacy of Biontech and Sinovac vaccines was reported to be 96.3% and 91.7%, respectively [37]. Similar to our study, the effectiveness of the Biontech vaccine was found to be higher. According to our study results, COVID-19 vaccine effectiveness is similar to other results of different populations. Both Biontech and Sinovac vaccines are found effective to prevent COVID-19 infection in Syrian refugees living in Istanbul. This situation is encouraging for vaccination programmes targeting Syrian refugees.

### Limitations and strengths

The vaccination status of individuals may be affected by some factors, such as educational and economical status, chronic diseases and vaccine hesitancy. These factors could not be evaluated in our study, which presents a limitation for the evaluation of our study results. Additionally, our study was not conducted as a population based study. It presents data of refugees who applied to a primary health care institution. However, it has been reported that a high percentage of Syrian refugees have difficulties in accessing health services [38]. The vaccination percentages may be lower in people who do not apply to a health institution. This fact is another limitation of our study.

There are many studies in the literature that evaluate the vaccination rates in different populations. However, data about vaccine effectiveness is limited, especially among refugees. While most of the studies evaluated the effectiveness of Biontech vaccine, we have evaluated the effectivenesses of both Sinovac and Biontech vaccines. This is the strength of our study. Moreover, in our study, we were able to evaluate also the data about COVID-19 infection according to age groups. Thus, all our results provide a broad perspective to the literature. This is another strength of the study. As we know; there is no study evaluating the COVID-19 vaccine effectiveness in Syrian refugees, so we think that our study creates novelty on this topic.

## Conclusions

Of the refugees in our study, more than 1 in 3 refugees were unvaccinated against COVID-19 disease. For Biontech and Sinovac vaccines, vaccine effectivenesses were found as 89.2% and 81.2%. respectively. The results of the study highlight the importance of vaccinations against COVID-19 pandemic, since both vaccines were highly protective against COVID-19 infection in our study. Although the effectivenesses of both vaccines were high, vaccination rates in refugees have been observed to be quite low in the study. Further research should be dedicated to investigating the obstacles hindering vaccination uptake among refugees. Since there are no studies on the COVID-19 vaccine effectiveness of Syrian refugees in our country, our study results will guide health interventions in this field. Several policy interventions and strategies can be implemented to enhance vaccine coverage among Syrian refugees. Since higher knowledge regarding the disease and vaccination provides positive attitudes [39]; community education, including training for healthcare providers and employing community health workers can help build trust and provide accurate information. Increasing accessibility through mobile vaccination clinics can be also useful for improving vaccination rates among refugees. Besides, vaccination services can be integrated with other health services to make them more accessible.

### Electronic supplementary material

Below is the link to the electronic supplementary material.

Supplementary Material 1

## Data Availability

No datasets were generated or analysed during the current study.
