# Supplementary material for: Frequency of SARS-COV-2 infection and COVID-19 vaccine uptake and protection among Syrian refugees: COVID-19 Vaccine among Syrian Refugees
Source: BMC Infect Dis. 2024 Jun 8;24:570. doi: 10.1186/s12879-024-09460-4 (PMC11161936; doi:10.1186/s12879-024-09460-4)
Supplement: Supplementary file 1 — Supplementary Material 1 [file 12879_2024_9460_MOESM1_ESM.docx]

**Supplementary Figure 2a.** COVID-19 vaccination doses and types (18-64 years)

**All the 4^th^ doses are Biontech vaccine.*

**Supplementary Figure 2b.** COVID-19 vaccination doses and types (65≥ years)
